# Making yogurt with the ant holobiont uncovers bacteria, acids, and enzymes for food fermentation

**DOI:** 10.1016/j.isci.2025.113595

**Published:** 2025-10-03

**Authors:** Veronica M. Sinotte, Verónica Ramos-Viana, Diego Prado Vásquez, Sevgi Mutlu Sirakova, Nabila Rodríguez Valerón, Ana Cuesta-Maté, Shannara K. Taylor Parkins, Julia Giecko, Esther Merino Velasco, David Zilber, Rasmus Munk, Sandra B. Andersen, Robert R. Dunn, Leonie J. Jahn

**Affiliations:** 1 Centre for Evolutionary Hologenomics, Globe Institute, University of Copenhagen, Copenhagen, Denmark; 2 Section for Food Microbiology, Gut Health, and Fermentation, Department of Food Science, University of Copenhagen, Copenhagen, Denmark; 3 Novo Nordisk Foundation Centre for Biosustainability, DTU Biosustain, Kgs. Lyngby, Denmark; 4 Alchemist Explore, Research and Development, Alchemist Aps, Copenhagen, Denmark; 5 Rachel Carson Centre for Environment and Society, LMU, Munich, Germany; 6 Basque Culinary Centre, Facultad de Ciencias Gastronómicas, Mondragon Unibertsitatea, Donosita-San Sebastián, Spain; 7 Food & Beverages Applied R&D, Novonesis, Hørsholm, Denmark; 8 Department of Applied Ecology, North Carolina State University, Raleigh, NC, USA

**Keywords:** Entomology, Food microbiology

## Abstract

Milk fermentation has a rich history in which food culture, the environment, and microbes intersect. However, traditional practices and their associated microbes have largely been replaced by industrial processes. We investigate a historical fermentation originating from Turkey and Bulgaria – ant yogurt. By examining the traditional practice, gastronomic applications, and experimentally derived yogurts, we uncover that the red wood ant holobiont facilitates fermentation. Bacteria hosted by the ants can proliferate in the milk. Specifically, live ants contribute lactic and acetic acid bacteria, including *Frutilactobacillus sanfranciscensis,* normally related to sourdough. Consequently, the bacterial community introduces lactic and acetic acid, while the ants provide formic acid, collectively advantageous for yogurt acidification and coagulation. Last, the ants and bacteria produce potential casein-active proteases that may further alter the yogurt texture. Our findings highlight the value of integrating traditional and biological frameworks to uncover the origins and applications of fermented food microbes.

## Highlights

• Research in ethnography, biology, and gastronomy reveals ants’ role in fermentation
• Ants contribute lactic and acetic acid bacteria to the yogurt
• Ants contain fermentative microbes, including *Fructilactobacillus sanfranciscensis*
• The ant holobiont contributes formic acid and proteases integral to fermentation

## Graphical abstract

**Figure:**
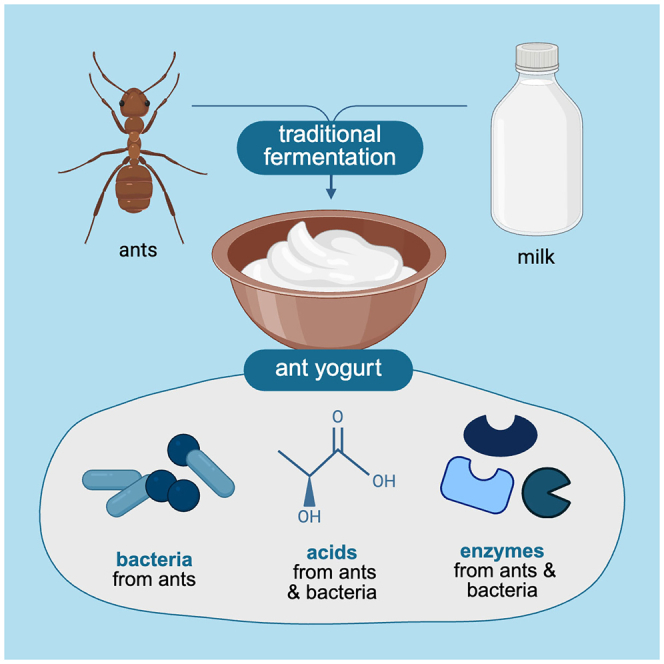

## Introduction

The fermentation of milk into products such as yogurt, cheese, and kefir originates from ancient practices and has dramatically shaped food cultures. The oldest archaeological evidence for dairying dates to 9,000 years ago in Anatolia (modern-day Turkey).^1^ Prehistoric dairy fermentation potentially occurred as early as 7,000 years ago, based on fat and protein residues isolated from ceramics resembling cheese strainers.^2^^,^^3^ In the millennia to follow, diverse dairy practices transformed milk into a preservable, widespread, and nutritious resource.^4^^,^^5^^,^^6^ Following suit, dairy fermentation became indispensable to regional cuisines and languages.^7^^,^^8^ Yogurt, a tangy fermented milk product, was thus a functional cultural adaptation dependent upon interactions between people, dairy animals, the environment, and most importantly, microbes. It is microbes that enter the milk, and through their enzymatic processes, catalyze the fermentation to acidic, viscous yogurt.^9^ These interspecies relationships are reflected in the Turkish word for a fermentation starter, *maya*, that ultimately “comes from relations within the broader web of life,” including microbes, animals, plants, and human culture.^7^

In the early 1900s, microbiologists characterized the first yogurt culture, laying the foundation for a pivotal shift from the diversity inherent in traditional yogurt to a simplified industrialized yogurt. Stamen Grigorov and Ilya Metchnikoff isolated and popularized a species of bacteria from Bulgarian *maya*, *Lactobacillus delbrueckii* subsp. *bulgaricus*.^10^^,^^11^ Following Metchnikoff, the industrialization of yogurt focused on a small number of bacterial taxa, predominantly *L. delbrueckii* subsp. *bulgaricus* and *Streptococcus thermophilus*.^12^ Both species are lactic acid bacteria, which play an important role in industrial and wild fermentations through food preservation, flavor generation, and potential health benefits.^13^^,^^14^^,^^15^ However, the focus on a few bacterial species overlooks the biodiversity embodied in traditional yogurts, which can include multiple species and strains.^16^^,^^17^ Revisiting biocultural origins of the yogurt fermentation offers opportunities to explore this biodiversity of starter cultures,^18^ to understand the interconnectedness of food systems, and to illuminate the history of yogurt and its cultural praxis.

The bacteria in yogurt stem from the multispecies dimensions of *maya.* Generally, the bacterial community that first colonizes the milk and establishes the fermentation ecosystem may stem from multiple environmental materials. Then, yogurt cultures are propagated by adding a small amount from a primary or old yogurt to fresh milk, a process referred to as “backslopping.” Environmental reservoirs that may inoculate microbes into the first ferment include the dairy animal,^19^ the person making the ferment,^20^^,^^21^ or the environment, such as vegetation,^22^ air, or containers used, as well as specific starter materials. For example, in the Turkish mountain villages of Kütahya and Eskişehir, yogurt fermentation is initiated by adding pinecones, which have been shown to introduce key microbial species, including *L. delbrueckii* and *S. thermophilus*.^23^ Other plant materials engaged to aid milk fermentation in Turkey and other countries include chamomile flowers, linden flowers, and nettle roots.^7^^,^^24^ In a broader context, leaves from plants such as nettle, fig, and butterwort, among others, have long been employed in traditional practices around the world to induce milk curdling and support subsequent fermentation.^25^^,^^26^^,^^27^ However, the environmental materials added to initate the first fermentation of yogurt are not limited to plants.

We aim to elucidate the biological catalysts underlying another traditional Turkish and Bulgarian yogurt-making practice - ant yogurt. This historic practice for starting the first or primary yogurt has been documented across the Balkan Peninsula and Turkey. Many of the practices in Turkey include ant eggs, larvae, pupae, or the surrounding nest material.^8^^,^^24^^,^^28^^,^^29^^,^^30^ For example, ethnographer Ali Rıza Yalman observed, “If the nomads want to make yogurt and cannot find enough starter culture to make yogurt, they crush the tiny eggs of the ants sheltering under the stones in their palms. When you put this into the milk […], that milk becomes yogurt.”^31^ Further oral histories from the Sharri mountains of Albania and North Macedonia also recall the use of ants in traditional yogurt fermentation (F. Demiraj, personal communication, May 2023). It is beyond the scope of our work, but the history and prehistory of these uses and their movement across Eurasia is a fascinating subject for future inquiry. Our focus is on a traditional spring practice from Bulgaria that involves fermenting yogurt within a red wood ant colony.^7^ This Bulgarian practice has been recently researched by our co-author (S. Mutlu Sirakova), and thus, it allows further exploration of red wood ants' contributions to fermentation. Finally, we note that these countries are connected by cultural threads preceding the establishment of national boundaries, contributing to general continuity between their culinary practices, both today and across millennia. The ethnographic evidence of ant yogurt across regions suggests ants may play an overlooked functional role in fermentation.

**Figure 1:**
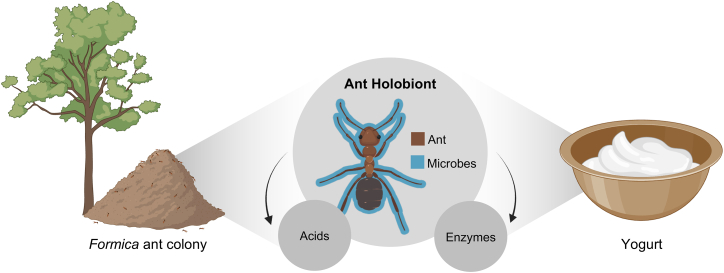
Hypothesized contributions of the ant holobiont to yogurt fermentation *Formica* red wood ants create characteristic thatched mounds (left), which may have been used in traditional yogurt fermentation practices.^7^ The traits of the ant holobiont, the composite of both ant and the microbes found within it, are hypothesized to act as the starter of the yogurt fermentation (center). These include the microbes (lactic acid and acetic acid bacteria) that are found in and on the ants, acids that are produced by the ant and the ant-associated microbes, and enzymes that have ant or microbial origins. This figure was created in BioRender and is under a CC-BY license (https://BioRender.com/enwovbv).

Here, we test the hypothesis that yogurt fermentation can be initiated by the ant “holobiont,” which includes both the ant and the microbial communities inherent to it.^32^^,^^33^ The ant holobiont, both ant and microbes, may contribute acids and enzymes key to the fermentation (Figure 1). First, we explored the potential of red wood ants (*Formica rufa* group)^34^ as starter cultures for yogurt based on ethnographic accounts and as culinary ingredients for modern gastronomy. Then, we characterized the microbial community of the ants *F. rufa* and *F. polyctena.* Yogurts derived from *F. polyctena* were made under controlled laboratory conditions to assess the contributions of the ant holobiont. These yogurts were prepared with live, frozen, or frozen then subsequently dehydrated ants. Live ants allow all microbes hosted by the ant to enter the fermentation. Freezing and dehydration are commonly used in culinary applications, because freezing kills a parasite the ants may carry,^35^ yet this treatment may also alter other living microorganisms. For these yogurts, we characterized the bacterial microbiome, quantified organic acids, and assessed the proteases and peptidases originating from ants and bacteria (Figure S1). Overall, the study examines an overlooked ecological niche of bacteria in the context of traditional practices potentially key to past and future fermented foods.

**Figure 2:**
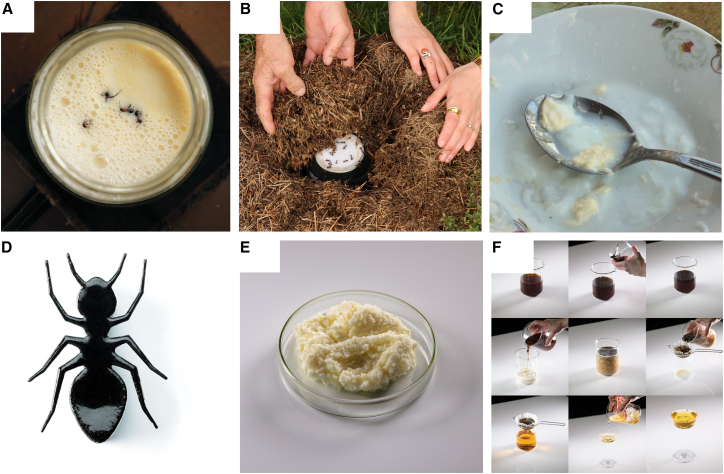
The ant holobiont serves as a catalyzing agent for dairy applications (A–C) Photographs of traditional ant yogurt fermentation initiated by *Formica rufa* ants and their characteristic thatched colonies, taken during field work in Bulgaria. (A) Live ants were added to warmed milk that was then (B) buried within the ant colony and left to ferment overnight. (C) The resulting milk had started to coagulate and acidify, indicative of early stages of yogurt fermentation. (D–F) Culinary applications created by the research and development team of two Michelin-star restaurant, Alchemist, using *F. rufa* ants: (D) ant yogurt ice cream sandwich, where the top view shows the tuile cookie that sandwiches the ice cream contained below (E) ant “mascarpone-like” cheese, and (F) milk-wash cocktail (Photos A-C by David Zilber, and D-F by Søren Gammelmark and Kåre Knudsen of Alchemist).

## Results

### The ant holobiont serves as a catalyzing agent for food applications

We worked toward a holistic understanding of traditional uses of ants as a yogurt starter by conducting fieldwork in Bulgaria in a community that retained an oral history of the practice.^7^^,^^36^ This community is also the ancestral home of one of the authors (S. Mutlu Sirakova). We used an ant colony selected by members of the community, which was the species *F. rufa*. This is one of the four species in the *F. rufa* group that maintain species ranges within Bulgaria.^37^ To make the yogurt, four live ants were added to a jar of warmed raw milk (Figure 2A), a cheese cloth was placed over the top of the glass jar, and the jar was left to ferment within the colony overnight (Figure 2B). Here, the ants could act as an inoculum, and the colony could serve as an incubator since the nest itself is known to produce heat.^38^ The jar was retrieved after a day of incubation. The acidity, texture, and flavor were indicative of early stages of yogurt fermentation. We observed that the milk had acidified to pH 5, coagulated at the bottom of the container (Figure 2C), and “had a slight tangy taste with mild herbaceousness and pronounced flavors of grass-fed fat” (D. Zilber).

Culinary applications of the *F. rufa* ants were created by the research and development team at restaurant Alchemist, ranked number eight in the world,^39^ holding two Michelin stars,^40^ and known for science-centered innovation. Co-authors based these applications on previous experiments where the addition of ants to milk caused coagulation. Presumably, this was due to the *F. rufa* ants' formic acid. Discussions on the traditional uses of ants in dairying (T. Tan, personal communication, April 2022) further directed the development of culinary applications. Thus, the innovations leverage the potential of ant-derived formic acid and microbe-derived organic acids to coagulate milk.

Three culinary applications were developed using live ants and ants that were frozen and subsequently dehydrated. First, the “ant-wich” contained ice cream derived from sheep yogurt made with live ants as starter culture (Figure 2D). The ice cream is sandwiched between an ant-infused gel and tuile cookies, and the sandwich is shaped as an ant with a laser-cut stencil. The ants provided a distinct, pungent acidity that contrasts with the fat of the milk, and a serving temperature of −11°C balances the desert. Second, a goat milk “mascarpone” was developed using dehydrated ants to catalyze the milk coagulation (Figure 2E). The texture was such as commercial mascarpone, yet the flavor was pungent and aromatic, similar to a mature pecorino cheese. Third, a milk wash cocktail was created, which is a dairy-based cocktail dating to the early 1700s.^41^ Classically, the milk is curdled with acid from citrus and subsequently filtered to remove the dairy solids, resulting in a clear beverage with more richness and body. In this case, dehydrated ants induced the curdling and separation of milk (Figure 2F). The cocktail had fruity notes from the apricot liqueur and the brandy (raisins, dried figs, and caramelized apples), and a silky texture from the residual milk whey. Replacing citrus with ants resulted in a milder acidity and a distinct flavor with additional fruity notes.

Despite the potential of these culinary innovations, we caution against their general application unless users are cultural practitioners or skilled food microbiologists. There are several food safety concerns that may arise. For example, the live ants may contain a parasite that can cause negative health outcomes for humans,^42^ although their prevalence is low.^43^ In culinary applications with live ants, the ants were crushed, mixed with a small amount of milk, and this was strained through a filter with a pore size of 100 μm. The filtration should remove any potential parasites, which are on average 332 × 221 μm,^35^ while allowing any bacteria or yeast to move through the filter into the ferment. Freezing has also been demonstrated to kill this parasite,^35^ and thus the ants are frozen for many culinary applications. However, freezing and subsequent prolonged incubation in warmer temperatures, such as those used in fermentation, may favor food-borne pathogens. Last, we note that ants are not included among the four insects authorized for sale as a food product in the European Union according to Regulation 2015/2283 on Novel Foods. The Novel Food regulations consider all foods not listed as being traditionally consumed in the EU prior to 1997 to be unauthorized. Thus, despite the traditional use of the ants documented here, they have yet to be acknowledged by this legislation.

Given that ants have diverse culinary applications with potential to initiate fermentation, both traditional and modern, we aimed to further elucidate the role of the ant holobiont. We hypothesized that the ant holobiont, consisting of the ant and its microbial partners, contributes to fermentation with bacteria, acids, and enzymes. To address this hypothesis, we created three elaborations of fermented milk with live, frozen, or dehydrated ants, per the modern gastronomic applications. The ants were collected in late spring and early autumn to further determine the impact of the season on the microbiome and the fermentation. While these experiments may not capture the breadth of multispecies contributors to traditional fermentations - ant, microbe, human, or other natural contributors—it is an essential first step to elucidate the biological mechanisms at play.

### Live ants provide stable and controlled yogurt microbiomes

**Figure 3:**
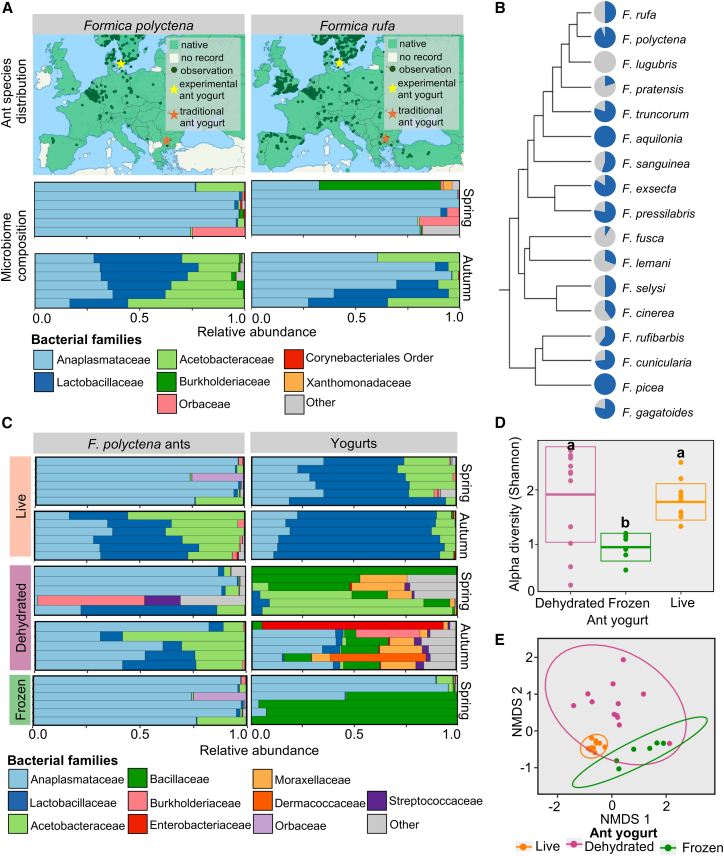
*Formica* ants consistently host lactic and acetic acid bacteria that contribute to stable yogurt microbiomes (A) European species ranges of *F. polyctena* and *F. rufa* (top), used within experimental and traditional ant yogurts, respectively (maps modified from antmaps.org). The microbiome composition of ant species across seasons (bottom). Bars represents four pooled ants from a colony of each respective species, collected in Denmark. The top 8 bacterial families among the samples are illustrated. (B) The prevalence of lactic acid bacteria (Lactobacillaceae; blue) in *Formica* across the ant phylogeny. Microbiome data are compiled from this study and previous research^48^^,^^49^^,^^50^^,^^51^; phylogenies are based on Jackson, Borowiec, and colleagues.^44^^,^^48^ (C) The bacterial microbiome of the three ant preparations and corresponding ant yogurts made in spring and autumn. Bars represent replicates, and the top ten bacterial families are shown. Live ant and frozen ant microbiomes are identical, as the live ants that were frozen prior to DNA extraction and thus are also representative of the frozen ants themselves. They are shown for direct comparison to the corresponding yogurts. (D) The alpha diversity (Shannon diversity) of ant yogurts. Letters indicate pairwise statistical differences (Tukey HSD: *p* < 0.05), the central line of the boxes indicates the median, and the edges of the boxes illustrate ± standard error measures. (E) The beta diversity of ant yogurts based on Bray-Curtis distances and Non-Metric Multidimensional Scaling (NMDS) analysis. The grouping is supported by a PERMANOVA (*p* < 0.0001).

We first characterized the bacterial microbiome of red wood ant sister species *F. rufa* and *F. polyctena*^44^ with 16S *rRNA* metabarcoding. Our aim was to examine how the ant yogurt practice may be translated across species and geographies. These ants have ranges spanning Denmark, where experimental yogurts were made, and Bulgaria or Turkey,^45^^,^^46^ where ethnographic histories originated (Figure 3A). Both species are found along the edges of pine forests, have thatched pine needle mounds, and near-identical morphological characteristics.^47^ These similarities make them all but indistinguishable in the field to ant experts,^47^ suggesting both may have been used in traditional and modern culinary applications.

The microbiomes of the two *Formica* species were dominated by lactic acid bacteria (Lactobacillaceae), acetic acid bacteria (Acetobacteraceae), and obligate intracellular bacteria (Anaplasmataceae) (Figure 3A). The bacterial families and genera (Figure S2A) align with *Formica*-specific microbiomes associated with species across the Northern Hemisphere.^48^^,^^49^^,^^50^^,^^52^ For example, *Formica* species across the clade host lactic acid bacteria of the genera *Lactobacillus* or *Fructilactobacillus* (Figure 3B). Thus, while there likely is colony-to-colony variation beyond that of the single ant colony represented here, the dominant bacteria are remarkably consistent with genus-level microbiome patterns. *Formica* ant microbiomes also appear to be seasonal. We observed an increase in the relative abundance of lactic and acetic acid bacteria and the bacterial biomass (i.e., load) from spring to autumn (Figure 3A; Figure S2B). Seasonal microbiomes have not been previously documented in *Formica* ants. If broader patterns across colonies, species, or populations agree with these initial results, it would suggest conserved seasonal microbiomes. Seasonal variation in the abundances of bacteria is germane to the use of these ants in fermentation because it may alter the number of bacteria in the starter. Overall, the consistent presence of lactic and acetic acid bacteria in the *F. rufa* group indicates the ant microbiome may be pertinent to food fermentation.

We then tested the hypothesis that lactic and acetic acid bacteria in the ants may transfer to the yogurt. Experimental ant yogurts were made under aseptic conditions in the lab with *F. polyctena* ants, where ants were live, frozen, or dehydrated. These yogurts aimed to discern the contribution of the ants to the fermentation observed in the traditional and culinary applications. We note that the fermentation may progress differently across species of ants, and we cannot definitively determine if the *Formica* sister species in this case have the same potential for fermentation. For example, the lactic acid bacteria co-evolved with the ants and presumably the slight differences in ant host biology across species. Regardless, this is the first step to determine if the lactic and acetic acid bacteria in the ants may contribute to the fermentation.

Based on 16S *rRNA* metabarcoding of experimental yogurts made with *F. polyctena* under aseptic conditions, we confirmed our hypothesis that bacteria from the ants contribute to the yogurt microbiome. The preparation of the ants distinctly impacted the bacterial communities in the resulting yogurts (Figure 3C). Like the ants themselves, the live ant yogurts were consistently dominated by lactic and acetic acid bacteria. Surprisingly, despite substantial seasonal differences in the ant microbiome, the corresponding yogurt microbiomes vary little in composition, suggesting yogurt fermentation may be possible regardless of variability in ant microbiomes. Here, the most abundant bacterial genus was *Fructilactobacillus* (Figure S2C). True abundances of *Fructilactobacillu*s may have been higher, considering our metabarcoding positive control indicates a slight underestimation of the abundance of lactic acid bacteria in samples (Figure S3). In contrast, dehydrated ant yogurts exhibited variable microbiomes. Frozen ant yogurts contained two bacteria groups: Bacillaceae, which can proliferate in the yogurt, and Anaplasmataceae, which obligately lives in ant cells^52^^,^^53^ and thus cannot grow in the yogurt. Here, dehydration and freezing likely reduced bacterial viability^54^ and consequently favored stochastic community assembly or freeze-resistant Bacillaceae.^55^ The persistence of lactic and acetic acid bacteria in live ant yogurt suggests it is the best starter for fermentation and provides biological support for the traditional, live ant-based yogurts.

We determined the consistency of the bacterial microbiome composition across the yogurts. Alpha and beta diversity metrics were used, where alpha diversity is the number of bacterial strains per sample (i.e., amplicon sequence variants) and beta diversity is the difference in the composition of strains from one sample to the next, weighting relative abundance. The ant preparations significantly affected the alpha and beta diversity of the yogurts (LM: F_2,27_ = 5.387, *p* = 0.0108, based on Shannon index; Adonis: F_2,27_ = 8.4402, *p* < 0.0001, based on Bray Curtis distances). The alpha diversity of the live ant yogurts was intermediate (Figure 3D), but the beta diversity was very low (Figure 3E). Therefore, the live ant yogurts tended to have the same species and composition from one sample/preparation to the next, as one would hope to see in predictable and controlled fermentations.

### Bacteria from the ants proliferate in the milk

**Figure 4:**
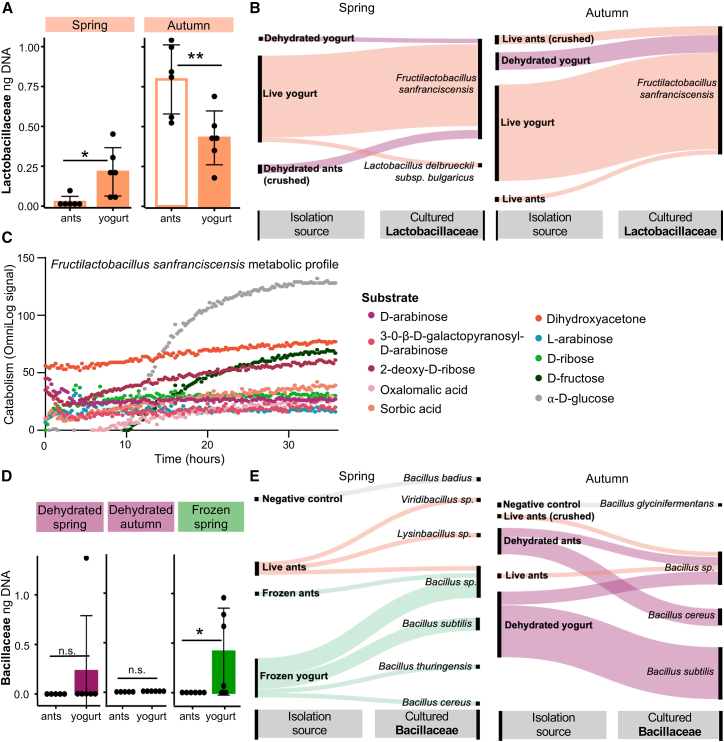
Bacteria from ants proliferate in the milk (A) The estimated amount of lactic acid bacteria (Lactobacillaceae) DNA, representative of lactic acid bacterial load, in the live ants and resulting yogurts across seasons. Dots indicate individual samples, and the data is represented as the mean ± standard error measures. The y-axis is presented on a logarithmic scale. (B) The diversity of culturable species of lactic acid bacteria from the ants and yogurts, where the majority of isolates stem from live ants and live ant yogurts. The thickness of each line approximates the abundance of culturable bacteria by representing the number of microbial media plates where the bacteria grew compared to other samples. (C) The metabolic potential of *F. sanfranciscensis* isolated from the ant yogurts. Catabolism of carbohydrate substrate arrays was measured over 36 h, where the strongest metabolic signal was observed for the ten compounds listed. (D) The amount of DNA from spore-forming Bacillaceae, indicative of Bacillaceae bacterial load, is exceptionally low in dehydrated and frozen ants and increases in the respective yogurt fermentations. Dots indicate individual samples, and the data are represented as the mean ± standard error measures. The y-axis is presented on a logarithmic scale. (E) Culturable species of Bacillaceae isolated from ants, yogurts, and negative control yogurts. Frozen ant yogurts were not made in autumn. Line thickness represents an approximation of culturable bacteria as above. (∗ = *p* ≤ 0.05; ∗∗ = *p* ≤ 0.01; ∗∗∗ = *p* ≤ 0.001; n.s. = *p* > 0.05).

We hypothesized that bacteria from the ant holobiont grew in the milk, facilitating fermentation. To assess growth, we determined the bacterial load in ants and yogurts with quantitative PCR (qPCR) and identified viable bacteria with culturomics using 16S *rRNA* Sanger sequencing. Live ants introduced lactic acid bacteria that proliferated in the yogurt. In the spring, yogurts contained higher loads of lactic acid bacteria than the ants introduced as a starter (Figure 4A; *t* (2, 10) = −2.975, *p* = 0.0139). However, in autumn, we observed the opposite pattern (Figure 4A; *t* (2, 10) = 3.269, *p* = 0.0084), suggesting that not all lactic acid bacteria from the ants enter the milk, or that the bacteria associated with the autumn ants are less prolific. While lactic acid bacteria dominated live and yogurt cultures, they made up a marginal amount of the bacterial load in frozen and dehydrated yogurts (Figure S4A).

Yogurt made with live ants contained several species of culturable lactic acid bacteria (Figure 4B). Aligned with the dominance of *Fructilactobacillus* in the community analysis, we isolated *F. sanfranciscensis*, a bacterium not only associated with ants^50^ but also sourdough bread fermentation.^56^^,^^57^ We further cultured an isolate of *Lactobacillus delbrueckii* subsp*. bulgaricus*, typically found in fermented dairy products (FAO & WHO,^12^ 2018). Last, we isolated the acetic acid bacteria, *Oecophyllibacter saccharovorans* (Figure S5), previously characterized in association with ant genera closely related to *Formica*.^58^^,^^59^^,^^60^ The diversity of culturable lactic acid bacteria thus indicates a niche overlap between ants and fermented foods.

Based on the prevalence (Figure 4B) and relative abundance (Figure S2A) of *Fructilactobacillus*, we characterized the metabolic potential of the isolated *F. sanfranciscensis*. The isolate was allowed to grow in carbon substrate arrays, and the catabolism of the individual substrates was quantified. The ant-associated *F. sanfranciscensis* catabolized ten different substrates (Figure 4C), where the most rapidly metabolized substrates were ɑ-D-glucose, D-fructose, and 2-deoxy-D-ribose. *F. sanfranciscensis* strains found in sourdough fermentations also can metabolize glucose, fructose, and ribose.^56^^,^^61^ The ant-associated strain can also break down dihydroxyacetone, which is known to be produced by the acetic acid bacteria also found in the ants and yogurt, *O. sacchoravorans*.^62^ This suggests that *F. sanfranciscensis* in ant yogurt metabolizes available sugars and acids, then lives off nucleic acid and proteins, contributing to the slower growth than conventional yogurt strains. Notably, we do not find metabolism of lactose, the main sugar in milk, nor sucrose and maltose, sugars typically used by sourdough strains.^56^ Therefore, within the ant niche, it may be unnecessary to maintain these metabolic functions. But in the process of adaptation to fermented food niches, the bacteria may gain these genes to metabolize carbohydrates in yogurt or sourdough. This potentially explains the differences between the bacteria assayed here and those common in ferments.

In the yogurts made with dehydrated and frozen ants, the bacterial load disproportionately came from spore-forming Bacillaceae. Notably, there is little to no Bacillaceae biomass in the ants, especially when compared to the yogurts (Figure 4D; *t*_frozen_ (2, 10) = −2.307, *p* = 0.0437), and this Bacillaceae did not proliferate in live ant yogurts (Figure S4B). We cultured several species of Bacillaceae, stemming largely from dehydrated or frozen ants or yogurts (Figure 4E). This included the food contaminant, *Bacillus cereus*.^63^ Although we cannot determine if the *Bacillus* load was sufficient to be problematic for consumption, it still indicates a potential risk. Therefore, we conclude that the microbiome of dehydrated and frozen ants and their corresponding yogurts is undesirable for food fermentations.

### Ants and bacteria contribute to the acidification of yogurt

**Figure 5:**
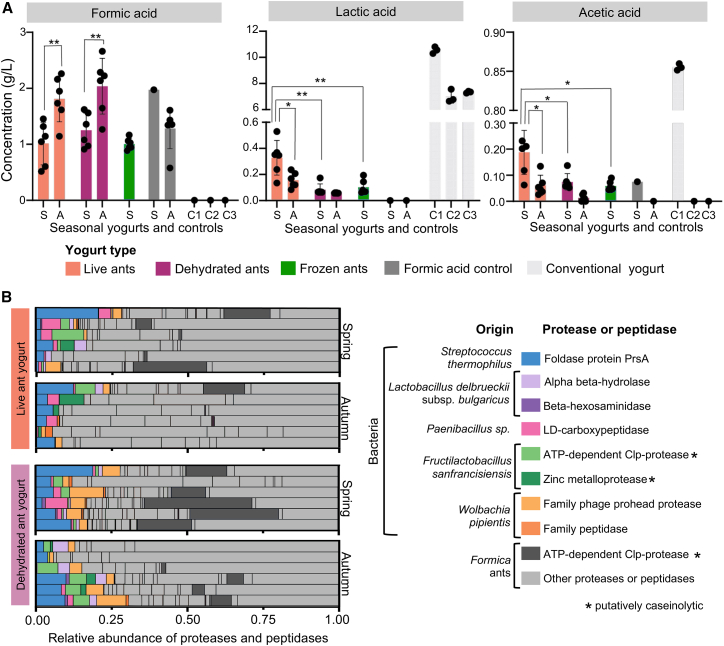
Organic acids, proteases, and peptidases in ant yogurts (A) Concentration of metabolites formic acid, lactic acid, and acetic acid in the yogurts from spring (S) and autumn (A). The samples include yogurts made with live, dehydrated, and frozen ants, formic acid, and three conventional yogurts as controls (C1-3). Data points represent biological replicates, and the data is represented as the mean ± standard deviation. Statistical analysis was performed using Student’s t-test (two-sample unequal variance; ∗ = *p* ≤ 0.05; ∗∗ = *p* ≤ 0.01; ∗∗∗ = *p* ≤ 0.001). (B) Proteases and peptidases detected in live and dehydrated ant yogurt samples. Proteases and peptides originate from the ant holobiont, including the ant and its bacteria. It also includes bacteria found in conventional yogurt that were potentially present in low abundances. The rows represent biological replicates. Milk proteins were removed to improve the visualization of the proteases and peptidases. Milk proteins made up 96.19 ± 1.07% of the samples. The absolute abundance of all proteins in the proteomes can be found Table S2.

We hypothesized that the ant holobiont, composed of both the ant and its microbes, may contribute acids key to fermentation. In yogurt, bacteria metabolize lactose and consequently produce high amounts of lactic acid and often low amounts of acetic and formic acid.^64^ These acids are essential to the tangy flavor, thick texture, and preservation of yogurt.^65^ Moreover, *Formica* ants (though, notably, not all ants) have a venom gland that contains largely formic acid that may be up to 10% the ant's body weight,^66^ although the whole ant contains a more complex mélange of chemicals.^67^ Thus, the acid from the ant holobiont may have three effects. First, it may engender yogurt tastes and textures, even without the effects of microbes. Second, it can create acidic conditions that favor acid-producing microbes. Third, the acid-producing microbes, independent of the ant, can themselves alter the acidity and flavor of the ferment. To investigate if the components of the ant holobiont, body and microbes, contribute organic acids to the yogurt, we quantified formic, lactic, and acetic acid in yogurts and controls by HPLC and measured pH before and after fermentation (Figure 5).

The ant holobiont contributed formic acid, which was the most abundant acid found in the yogurts (Figure 5A). The amount of formic acid was in a similar range to that in the control, in which formic acid alone was added to pH 4.6 to induce coagulation. The season, but not the type of yogurt (live, dehydrated, frozen), further dictated the formic acid levels (LM results). The higher levels in autumn (t-test: *t*_live_ (2, 10) = −3.500, *p* = 0.0057; *t*_dehydrated_ (2, 10) = −3.297, *p* = 0.0081) may be due to the seasonality of formic acid production.^66^ Therefore, the acid from the ants entered the milk and likely shaped the unique acidic flavor and texture of the culinary applications, contrasting the rounded flavor of lactic acid typically predominant in fermented dairy.

Lactic and acetic acid were also detected in the ant yogurts, validating that ant-associated bacteria likely contributed acids during fermentation (Figure 5A). Acid profiles align with the microbiome data. Lactic and acetic acid bacteria were the most abundant genera in live ant yogurts (Figure 3A). Similarly, these acids are highest in live ant yogurts compared to dehydrated (*t*_lactic_ (2, 10) = 4.270, *p* = 0.0052; *t*_acetic_ (2, 10) = 3.065, *p* = 0.0220) and frozen yogurts (*t*_lactic_ (2, 10) = 3.842, *p* = 0.0060; *t*_acetic_ (2, 10) = 3.653, *p* = 0.0147). In live yogurts, we observed that lactic and acetic acid were higher in spring (*t*_lactic_ (2, 10) = 2.897, *p* = 0.0231; *t*_acetic_ (2, 10) = 3.325, *p* = 0.012), the season in which we isolated the greatest diversity of lactic acid bacteria (Figure 4C). This suggests different strains of bacteria may inherently vary in acid production. Notably, ant yogurts had less lactic acid, acetic acid (Figure 5A), and bacterial biomass (Figure S6A) than conventional or homemade yogurts. We did not establish and perform the optimal fermentation conditions for the ant yogurt, and thus, the fermentation was incomplete. However, we can conclude that the ant microbes contribute organic acids to fermentation.

Ant yogurts contained, on average, 2.5 g/L total organic acids, while conventional yogurts contained up to 12 g/L total organic acids. Thus, the ant yogurt remained in a pH range of 5.0–5.9 and did not reduce to a pH of 4.2 of conventional yogurt (Figure S6B), given the conditions and timing of our experiment. Initially, the pH of the milk dropped upon the addition of ants, likely due to the formic acid from the ants (Table S1). The pH continued to decrease through the fermentation (Figure S6B), likely because of acid-producing microbes. Typically, in yogurt fermentation, a drop to pH 5.3 initiates coagulation, and a further acidification to pH 4.6 completes coagulation by causing the aggregation of casein micelles.^68^ The ant yogurt coagulated yet largely did not reach such a low pH. This suggests another aspect of the fermentation denatured caseins, namely proteases.

### Ants and microbes contribute proteases and peptidases, potentially modifying yogurt texture

Our final hypothesis was that the ant holobiont may contribute enzymes, in particular proteases, that modify the yogurt texture. Proteases can differentially cleave and degrade casein, the main protein in milk, and result in a firm or fluid yogurt texture.^69^^,^^70^ Based on the pH and coagulation results, we predicted the ants themselves may contain milk-clotting proteases, such as those previously identified in mealworms.^71^ Casein proteases and peptidases are also typically produced by the bacteria in yogurt.^69^ Thus, the ant holobiont, ants and bacteria, may contribute proteases to the yogurt fermentation. To test this hypothesis, we conducted an untargeted proteomics analysis based on a self-curated database that contained proteases from *Formica* ants, ant-yogurt bacteria, conventional yogurt bacteria, and milk proteins.

We confirmed our hypothesis that the ant holobiont contributes proteases and peptidases to the yogurt fermentation (Figure 5B). Several ant proteases were identified in the yogurt (Figure 5B), one of which, an ATP-dependent CLP protease, is known to have caseinolytic potential.^72^ Ant-associated bacteria further contributed proteases and peptidases. The lactic acid bacteria *F. sanfranciscensis* produced two proteases, ATP-dependent CLP protease and zinc metalloprotease, both known to have caseinolytic homologues.^72^^,^^73^ Proteases and peptidases typically belonging to bacteria in conventional yogurt, *L. delbruecki*i subsp. *bulgaricus* and *Streptococcus thermophilus*, were found, as well as ant yogurt bacteria *Paenibacillus* and *Wolbachia pipentis*. While the presence of proteases suggests a potential role in the texturization of yogurt, their enzymatic activity and specific impact on the final yogurt texture would need to be investigated in greater detail to accurately determine their contribution.

Last, we identified milk proteins such as casein, which made up a high percentage 96.19 ± 1.07% of the proteome of samples (Table S2), potentially because of the low bacterial load compared to conventional yogurt. There were no significant differences in the relative abundance of all proteins in the proteome between live and dehydrated ant yogurts or seasons (Figure S7). Thus, proteases in both live and dehydrated yogurts may, in part, catalyze coagulation in the experimental yogurts and in the modern culinary applications. Taken together, the ant holobiont, ants and microbes, contribute proteases potentially relevant to food fermentation.

## Discussion

In this study, we explored the traditional practice of using ants as a starter for yogurt fermentation. We hypothesized that the ant holobiont, both the ant and its microbes, catalyzed the fermentation. First, we uncovered bacteria from the *F. polyctena* ants that shaped the yogurt microbiome. Using live ants as a starter, in contrast to frozen or dehydrated ants, resulted in a consistent microbiome containing lactic and acetic acid bacteria, desirable for fermentation. These ant-bacteria included some species thought of as canonical fermentation microbes, such as *F. sanfranciscensis*. Second, we discovered that the addition of the ant holobiont to milk introduced acids and proteases. Formic acid originated from the ant, while lactic and acetic acid were likely produced by bacteria. The ants and bacteria also contributed proteases with caseinolytic potential. Our results revealed that the ant holobiont can contribute to fermentation and thus prompted the reconsideration of the microbial consortia in our ferments, their origins, and applications to fermented food.

We uncovered how ants and their bacteria synergistically shape the fermentation by applying the holobiont framework. Holobiont theory considers a host and its associated microbes collectively, where the interplay between animal and microbes shapes the resulting ecology and evolution of the whole, host plus microbes.^32^^,^^33^^,^^74^ When animals or plants are used in fermentation, their holobionts may structure that fermentation in ways that reach beyond the effect of the individual parts. In this study, it was thus essential to include the entire ant holobiont, rather than isolating the bacteria alone for fermentation. *Formica* ants produce formic acid for defense^66^^,^^75^ and feed on the sugar-rich honeydew^76^ and protein-rich insects.^34^ In response, the lactic and acetic acid bacteria are hypothesized to metabolize the honeydew,^50^ are highly acid tolerant^75^ and are potential contributors to proteolysis. Once ants are introduced into milk, the properties of the ant holobiont extend to the yogurt ecosystem. Formic acid initially acidifies the milk, ant-bacteria break down milk sugars producing additional acid, and proteases liberate peptides and amino acids for bacterial growth. By looking through the lens of the holobiont, we could clarify why *F. polyctena* ants and bacteria are pre-adapted for the fermentation of yogurt and potentially other foods.

The effect of the ant holobiont on yogurt fermentation parallels the role of microbes in other fermented foods. In most ferments, multiple species of microbes offer complementary contributions to the fermentation process.^77^^,^^78^ In conventional yogurt, the bacteria *S. thermophilus* produces formic acid that is then metabolized by *L. delbrueckii* subsp. *bulgaricus*, and in return, *L. delbrueckii* subsp. *bulgaricus* produces proteases*.* Collectively, these bacteria then metabolize lactose and produce lactic acid to transform milk into tangy yogurt.^64^^,^^79^ In sourdough, yeast and lactic acid bacteria can secrete factors that promote one another’s growth,^80^ and overall have complementary roles in the fermentation of sugars and protein derivatives.^56^^,^^81^ Together, sourdough microbes create leavened bread with acidic, kokumi, and umami flavors.^56^ In the ant yogurt, our results suggest the separate elements of the ant holobiont, the ant body and microbes, act synergistically. Consequently, the ant holobiont introduces acids, proteases, and potentially other compounds that contribute to the organoleptic characteristics such as distinct acidity, coagulation, and flavor.

*Formica* ants host greater bacterial diversity that may hold wider purchase for fermented foods, beyond that of the ant species (*F. polyctena*) and strain we examine here. The bacterial species isolated most frequently from experimental yogurts, *F. sanfranciscensis,* is one of the most prevalent bacteria in sourdough bread globally.^82^^,^^83^ The *Fructilactobacillus* clade appears to have diversified with ants,^48^ suggesting these ants host multiple species and strains. For example, our results indicate that the ant-associated strain we examine lacks certain metabolic functions found in sourdough or yogurt. This suggests that there may be greater functional diversity in the bacteria hosted by *Formica* ants, or that the bacteria later adapted to niches outside of ants, such as fermented foods. In the future, the bacterial diversity hosted by these ants may be assayed for key functions in fermentation, such as sugar metabolism, volatile compound production, and exopolysaccharide formation. Depending upon these functions across substrates, we can determine which ferments these bacterial strains may be applicable to, such as sourdoughs or plant-based yogurts. Ultimately, these results lay the groundwork for unveiling the diversity of fermentative bacteria residing in ants and for elucidating their potential for the fermentation of different food substrates.

Our results bring forth questions we believe are central to understanding the ecological and ancient origins of fermentative microbes. The ecological origins and assembly of ferment microbiomes, which colonize fermentations in lieu of backslopping, are only beginning to be characterized.^84^ Recently, it has been convincingly argued that the wine yeasts can originate from social wasps, where the symbiosis between wasps and yeasts is essential for the microbial transfer to wine ecosystems.^85^^,^^86^^,^^87^ Similarly, our results are reconcilable with the idea that *F. sanfranciscensis* has evolved in ants over millions of years and was only introduced into fermented foods with the advent of bread making, which occurred in the last several thousand years.^88^^,^^89^ Beyond traditional practices using ants in yogurt,^7^^,^^8^^,^^31^ ant colony materials may be integrated in sourdough fermentation,^7^ providing further avenues for microbial transfer. Conceivably, the ant bacteria then could be backslopped in traditional fermentations, successively propagating and evolving in yogurts or sourdoughs. Although it is not clear when these traditions arose, it is noteworthy that the earliest evidence of yogurt and architectural depictions of ants and bees were found in prehistoric remains from our study region.^90^^,^^91^ While these fragmentary anecdotes do not reconcile or resolve the origins of fermentative microbes, they highlight cultural threads that may connect ants and ferments.

While ant yogurt highlights the significance of biocultural interactions, we caution against its production unless practitioners maintain it as part of their heritage or have extensive experience in food microbiology. First, *Formica* red wood ants are of conservation concern due to recent declines in populations,^92^ and wide scale collection is not sustainable. The bacteria within these ants, which can be isolated and used separately for fermentation, underscore the value of this biodiversity. Furthermore, ant yogurt carries several food safety risks and thus requires specialized knowledge. *Formica* ants carry a parasite, *Dicrocoelium dendriticum*, which occurs in low prevalence but can have negative health outcomes for humans.^42^^,^^43^ In culinary applications with live ants, we strained the ant starter with a microbiology-grade sieve to remove any potential parasites. In modern gastronomy, *Formica* ants are typically frozen or placed in alcohol to kill this parasite.^35^ However, we found that fermentation started with frozen ants promotes the growth of unfavorable bacterial communities, including potential food-borne pathogens such as *Bacillus* spp. Finally, *Formica* ants are considered a Novel Food by the European Union, despite evidence of culinary traditions, and thus are not approved for sale. In the traditional ant yogurt, knowledge holders may have maintained practices to prevent the spread of this parasite and food-borne pathogens. For example, we observed that the ants were not crushed in the traditional practice, which would prevent the release of the bacteria. Additionally, food safety cultures can limit risks such as *Bacillus* proliferation, which may also be avoided as ants are not frozen in the traditional practice. This poses intriguing questions about the intersection of food safety and traditional knowledge that are beyond the scope of this research. Thus, careful consideration of both the cultural and biological aspects of traditional or historical practices is paramount when exploring their re-creation.

As a group of co-authors, including anthropologists, culinary innovators, and food scientists, we differentially perceive the implications of the ant holobiont on yogurt-making. From an anthropological viewpoint, it challenges the ethos of conventional yogurt fermentation. Unlike traditional yogurts, large-scale industrial yogurt production relies on a limited number of bacterial strains. Conversely, the lactic acid bacteria found in ant yogurt are maintained in the ants, yogurt, and environment, and are cultured through traditional dairy practices passed down over generations.^7^ To preserve this microbial diversity, we must uphold its cultural heritage. From the food science perspective, microbes or enzymes from ant yogurts can be integrated into the food system to explore potential applications and flavors. For example, these ant microbes hold potential promise for plant-based foods, such as dairy-free yogurts. In relation to modern gastronomy, beyond the possibility of imagining sour or effervescent ferments, exposing the public to familiar foods made with microbes and insects could conceivably help change consumer perceptions of entomophagy and microbiology. While we differ in what the future of ant holobiont-facilitated fermentation may be, we agree it provides a powerful example that can broaden our thinking, as a society, about what is possible.

### Limitations of the study

This study shines light on the relation between traditional practices and microbial diversity, which together hold potential for advancing food science, elevating gastronomy, and embracing heritage. However, this work still has limitations. First, the study examines the potential of one *Formica polyctena* colony in fermentation in both the spring and autumn. Although the microbiome is consistent with other *Formica* ants, containing lactic and acetic acid bacteria including *Fructilactobacillus sanfranciscensis*, the fermentation itself may vary slightly with natural intracolony variability. Second, we did not clarify the optimal conditions for the fermentation, either in the lab or in traditional practice. Rather, this study highlights ants as a reservoir of bacteria with potential for food fermentation, and the importance of both ant biodiversity and traditional practices in maintaining this potential. Third, the study explores the traditional practice of one village. However, it remains clear that the use of ants in yogurt is widespread in Turkey and other Balkan countries, raising questions about the intricacies of the biocultural practice and its historical roots. Fourth, there are legitimate concerns about the consumption of live *Formica* ants, given that the ants may carry a parasite dangerous to humans. Therefore, we caution against the general application of this fermentation, unless a) practitioners maintain this as part of their heritage and cultures of food safety, or b) practitioners have knowledge in food microbiology to allow for adequate food safety.

## Resource availability

### Lead contact

Requests for further information and resources should be directed to and will be fulfilled by the lead contact, Leonie J. Jahn (lejj@biosustain.dtu.dk).

### Materials availability

This study did not generate new unique reagents.

### Data and code availability

• Amplicon sequences from metabarcoding of ants and yogurts are available on SRA (BioProject PRJNA1162421).
• Sanger sequences of isolated bacterial strains (PQ535580-PQ535692 and PQ651799-PQ651879) and ants (PQ453570-PQ453574) are available on GenBank.
• Proteome data will be made available on the ProteomeXchange database under identifier PXD057843.
• Additional data frames have been deposited on FigShare (https://doi.org/10.6084/m9.figshare.27918531).
• The code for analyses is available on Zenodo (https://doi.org/10.5281/zenodo.14626911).
• Any additional information required to reanalyze the data reported in this article is available from the lead contact upon request.

## Acknowledgments

We thank Rasmus Stenbak Larsen for the assistance in collecting the ant colonies. We acknowledge myrmecologist Dr. Vera Anatova from the Bulgarian Academy of Sciences, who provided knowledge and assisted us with the permit НС ЗП-122/18.04.2023 from the Ministry of Environment and Water to collect wood ants in Bulgaria. We thank villagers from Nova Mahala for sharing their knowledge, especially Musserem, Mehmet, and Fatma. We acknowledge gastronomers Fejsal Demiraj and Tangör Tan for their personal communications around the traditional use of ants in dairy fermentations. Last, we thank the laboratory staff at the Globe Institute for their expert support and guidance, especially Lasse Vinner, Tina Blumensaadt Brand, and Pernille Selmer Olsen. The Graphical Abstract was created in BioRender by VMS (2025; https://BioRender.com/miupvih). V.M.S., A.C.M., J.G., and S.B.A. were supported by the 10.13039/501100001732Danish National Research Foundation (Denmark) Center for Evolutionary Hologenomics: 10.13039/501100001732DNRF 143. V.R.V, and L.J.J. were supported by the 10.13039/501100009708Novo Nordisk Foundation (Denmark), NNF Grant number: NNF20CC0035580.

## Author contributions

Conceptualization: V.M.S., L.J.J., R.R.D., V.R-V., D.P.V., and S.M.S; methodology: V.M.S., V.R.-V., D.P.V, N.R.V, S.K.T.P, J.G., E.M.V, and S.B.A; formal analysis: V.M.S., V.R.-V., and S.K.T.P; investigation: V.M.S., V.R.-V. D.P.V., S.M.S., N.R.V., A.C-M., S.K.T.P., J.G., E.M.V., and D.Z.; resources: L.J.J., S.B.A, R.M., and S.M.S.; data curation: V.M.S.; writing – original draft preparation: V.M.S., V.R-V., L.J.J., and R.R.D; writing – review and editing: all authors; visualization: V.M.S. and V.R.-V.; supervision: L.J.J., R.D.D., S.B.A., and R.M.; project administration: V.M.S. and L.J.J.; funding acquisition: S.B.A., L.J.J., R.R.D., and R.M.

## Declaration of interests

L.J.J. is the founder of MATR Foods Aps. R.M. is the founder and owner of both restaurant Alchemist and Spora Aps.

## STAR★Methods

### Key resources table

**Table:** 

REAGENT or RESOURCE	SOURCE	IDENTIFIER
**Biological samples**		

*Formica rufa* ants	Nova Mahala, Bulgaria	41.5631, 24.16439
*Formica rufa* ants	Bøllemosen, Denmark	55.827676, 12.534667
*Formica polyctena* ants	Rude Skov, Denmark	55.8334720, 12.47493841
Microbial community standard	Zymobiomics, Nordic Biosite, Denmark	Cat no. Biosite-D6300

**Chemicals, peptides, and recombinant proteins**		

7% fat sheep’s milk	Hårbølle Mejeri, Møn, Demark	https://www.harbollemejeri.dk/
36% fat sheep’s cream	Hårbølle Mejeri, Møn, Demark	https://www.harbollemejeri.dk/
Sugar	Nordic Sugar, Denmark	https://www.nordzucker.com/en/
Gelatine sheets bloom 220	Condi, Denmark	Cat no. 10701
Ice cream stabilizer	Condi, Denmark	Cat no. 10770
Powdered glucose 33DE	Sosa, Spain	Cat no. 50053
Inverted sugar	Trimoline, Cristalco, France	https://cristalco.com/solutions/softcrisp/
NH pectin	Sosa, Denmark	Cat no. 41476
Sweetened condensed milk	Tørsleffs, Denmark	https://www.torsleffs.dk/produkter/kondenseret-maelk/
Wheat flour	Condi, Denmark	Cat no. 63893
Baking powder	Condi, Denmark	Cat no. 12219
Maldon Salt	Condi, Denmark	Cat no. 18166
Charcoal-black colorant	Sosa, Spain	Cat no. 39445
Brandy	Ximenex Spinola Battonage Brandy, Spain	https://ximenezspinola.com/products/brandy-battonage
Genepi liqueur	Genepi Grand Tetras, France	https://www.giffard.com/
Apricot liqueur	Bitter Truth Apricot Liqueur, Germany	https://the-bitter-truth.com/liqueurs-spirits/apricot-liqueur/
Chelex	Sigma Aldrich, Denmark	CAS 11139-85-8
Milk powder	Sigma Aldrich, Denmark	CAS 999999-99-4
Sodium citrate	Sigma Aldrich, Denmark	CAS 6132-04-3
Tris	Sigma Aldrich, Denmark	CAS 77-86-1
EDTA	Sigma Aldrich, Denmark	CAS 60-00-4
Sucrose	Sigma Aldrich, Denmark	CAS 57-50-1
Lysozyme	Sigma Aldrich, Denmark	Cat no. SAE0152
Mutanolysin	Sigma Aldrich, Denmark	Cat no. SAE0092
De Man, Rogosa and Sharpe (MRS) Media	VWR, Denmark	Cat no. 1.10661.0500
Gifu Anaerobic Mediua (GAM)	HyServe GmbH & Co.KG, Germany	Cat no. 1005433-001
Red Taq Mastermix	VWR, Denmark	Cat no. 733-5244
Formic acid	Sigma Aldrich, Denmark	CAS 64-18-6
Trifluoroacetic acid	Sigma Aldrich, Denmark	CAS 76-05-1
Acetonitrile	Sigma Aldrich, Denmark	CAS 75-05-8

**Critical commercial assays**		

Sanger Sequencing	Macrogen, Netherlands	https://order.macrogen-europe.com/main.do
Sanger Sequencing	Eurofins Scienfic, Sweden	https://www.eurofins.com/
PureIT ExoZap	Amplicon, Denmark	Cat no. A620601
DNeasy Blood and Tissue Kit	Qiagen, Denmark	Cat no. 69504
0.1 mm G2 DNA/RNA Enhancer Beads	Amplicon, Denmark	Cat no. A420150
AMP Pure XP Reagent	Beckman Coulter, Denmark	Cat no. A63881
DNeasy PowerClean Pro kit	Qiagen, Denmark	Cat no. 12997-50
Femto Bacterial DNA Quantification Kit	Zymobiomics, Nordic Biosite, Denmark	Cat no. Biosite-E2006
16S rRNA V3-V4 metabarcode sequencing	Novogene, Europe	https://www.novogene.com/eu-en/
Rapid Digestion Trypsin/Lys-C kit	Promega, Denmark	Cat no. VA1061
PMI Biolog carbohydrate substrate array	Biolog, Hayward, California, USA	Cat no. 12111
PM2 Biolog carbohydrate substrate array	Biolog, Hayward, California, USA	Cat no. 12112

**Deposited data**		

*Fructilactobacillus sanfranciscensis* proteome	Uniprot	UP000001285
*Oecophyllibacter saccharovorans* proteome	Uniprot	UP000315037
*Paenibacillus* sp proteome	Uniprot	UP000003445
*Wolbachia pipentis* proteome	Uniprot	UP000175679
*Streptococcus thermophilus* proteome	Uniprot	UP000001170
*Lactobacillus delbrueckii* subsp. *bulgaricus* proteome	Uniprot	UP000001259
*Bos taurus* proteome	Uniprot	UP000009136
Amplicon sequences from metabarcoding of ants and yogurts	This paper	SRA: BioProject PRJNA1162421
Sanger sequences of isolated bacterial strains	This paper	GenBank: PQ535580-PQ535692 and PQ651799-PQ651879
Sanger sequences from ants	This paper	GenBank: PQ453570-PQ453574
Proteome data from ant yogurts	This paper	ProteomeXchange: PXD057843
Additional data frames have been deposited on FigShare	This paper	https://doi.org/10.6084/m9.figshare.27918531
The code for analyses is available on Zenodo	This paper	https://doi.org/10.5281/zenodo.14626911

**Oligonucleotides**		

LCO1490 5′-GGTCAACAAATCAT AAAGATATTGG-3′	Zuccon et al.^93^	https://doi.org/10.1071/Is12027
HCO2198 5′-TAAACTTCAGGRTG ACCAAAAAATCA-3′	Zuccon et al.^93^	https://doi.org/10.1071/Is12027
341F 5’ -CCTAYGGGRBGCASCAG- 3′	Novogene, Europe	https://www.novogene.com/eu-en/
806R 5’ -GGACTACNNGGGTATCTAAT- 3′	Novogene, Europe	https://www.novogene.com/eu-en/
cPCR-F 5′-AGAGTTTGATCCTGGCTCAG-3′	Cebeci and Gürakan^94^	https://doi.org/10.1017/S0022029908003543
cPCR-R 5′-CCGTCAATTCCTTTRAGTTT-3′	Cebeci and Gürakan^94^	https://doi.org/10.1017/S0022029908003543

**Software and algorithms**		

Geneious Prime v.2024.0	Biomatters Ltd, New Zealand	https://www.geneious.com/
R v.4.3.1	Team^95^	https://www.R-project.org
dada2 v.1.30.0	Callahan et al.^96^	https://doi.org/10.1038/Nmeth.3869
SILVA database v.138.1	Glöckner et al.^97^	https://doi.org/10.1016/j.jbiotec.2017.06.1198
SCRuB v.0.0.1	Austin et al.^98^	https://doi.org/10.1038/s41587-023-01696-w
Phyloseq v.1.45.0	McMurdie and Holmes^99^	https://doi.org/10.1371/journal.pone.0061217
Microviz v.0.12.0	Barnett et al.^100^	https://doi.org/10.21105/joss.03201
ggplot2 v.3.3.3	Wickham^101^	https://doi.org/10.1002/wics.147
vegan v.2.6.4	Oksanen et al.^102^	10.32614/CRAN.package.vegan
parwiseAdonis v.0.4.1	Martinez^103^	https://github.com/pmartinezarbizu/pairwiseAdonis/tree/master/pairwiseAdonis
Chromeleon 7	Thermo Fisher Scientific, USA	https://www.thermofisher.com/order/catalog/product/CHROMELEON7
GraphPad Prism	GraphPad Software, Inc.	https://www.graphpad.com/features
Spectronaut v. 18	Biognosys, Switzerland	https://biognosys.com/software/spectronaut/
Omnilog PM software	Biolog, Hayward, California, USA	https://www.biolog.com/products/omnilog/

**Other**		

Corning cell strainer pore size 100 um	Sigma Aldrich, Demark	Cat no. CLS431752
0.2 μm syringe filter	Sigma Aldrich, Denmark	Cat no. SLLG025SS
Bio-Rad Aminex HPx87 column	Bio-Rad, USA	Cat no. 1250140
Empore C18 resin	Sigma Aldrich, Demark	Cat no. 6883-U

### Experimental model and study participant details

This study examines the microbiomes of two ant species, *Formica rufa* and *Formica polyctena*. Traditional yogurts, culinary applications, and experimental yogurts are made from these species. The ants were collected in Denmark and Bulgaria, at locations listed in the key resources table and Methods. Where necessary, permission was obtained for collection of ants; therefore, Bulgarian ants were collected under permit НС ЗП-122/18.04.2023 from the Ministry of Environment and Water.

### Method details

#### Traditional and modern culinary applications of ants

We traveled to Nova Mahala, Bulgaria to recreate ant yogurt with communities that retained memories and oral histories of the ant yogurt tradition. We based the yogurt fermentation on freshly acquired raw cow milk and *F. rufa* ants, from a colony found just outside of the village. The milk was warmed until nearly scalding, such that it could “bite your pinkie finger”.^7^ Four live *F. rufa* ants were added to 400 mL of milk. A cheesecloth was secured over the milk, and fabric was wrapped around the glass container for insulation. The milk jar was then buried inside the ant colony, covering it completely with the mound material. The nest itself is known to produce heat,^38^ and thus may act as an incubator for the yogurt fermentation. The milk was left within the colony and retrieved the next day, 26 h later. The yogurt was then tasted, checked with pH strips, and stirred to observe coagulation.

Three culinary applications of the *Formica rufa* ants were developed by the research and development team at Restaurant Alchemist.

##### Ant yogurt ice cream sandwich

The “ant-wich” consisted of ant yogurt ice cream, ant gel filling, and ant tuile. To make the ant yogurt ice cream, organic 7% fat sheep’s milk (Hårbølle Mejeri, Møn, Denmark) was fermented with 1.6% live ants. Live ants were crushed and mixed in an aliquot of milk, which was passed through a strainer with pore size 100 μm (Sigma Aldrich, Denmark) to remove ant body parts and potential parasites that may occur in low prevalence.^35^ The strained aliquot was mixed with the rest of the milk, followed by an incubation period of 8 h at 40°C and subsequent storage at 4°C overnight. To sweeten and thicken the yogurt into ice cream, 300 g of sheep’s cream (Hårbølle Mejeri Møn, Denmark), and 22 g of sugar (Nordic Sugar, Denmark) was added to 380 g of ant yogurt, followed by 3 g of gelatine sheets (Condi, bloom 220, Denmark) and 5 g of ice cream stabilizer. The ant gel filling, based on an ant infusion, was made for the ice cream sandwich. For the infusion, dehydrated ants (*Formica rufa*) and water (filtered, pH 7) were mixed at the ratio of 10/100 (v/w), vacuum infused at 85°C for 8 h, strained through a fine mesh, and kept at 4°C. Subsequently, the ant gel was made with 100 g of ant infusion, 7.5 g of powdered glucose 33DE (Sosa, Spain), 70 g of inverted sugar (Trimoline, Cristalco, France), and 5 g of NH pectin (Sosa, Denmark). These were mixed, cooked for 5 min, and set to rest at 4°C for 24 h. Finally, an ant tuile was made to sandwich the ant ice cream and filling. The tuile was made by mixing 100 g of sweetened condensed milk (Tørsleffs, Denmark), 100 g of 00 wheat flour (Condi, Denmark), 100 g of egg whites, 3 g of baking powder (Condi, Denmark), 2 g of salt (Maldon), and 8 g of charcoal-black colorant (Sosa, Spain). The mixture was blended for 5 min, spread to measure 2 mm in height, and baked in the oven at 155°C for 15 min. The final “ant-wich” was assembled to take the shape of an ant using a laser-cut stencil and served at −11°C.

##### Ant mascarpone-like cheese

A mascarpone-like cheese was developed using ants as the coagulant. In common practice, lemon juice and milk or cream is added to make mascarpone, where the citric acid coagulates the dairy. Here, the acid from the ants likely contributed to the coagulation. Pasteurized sheep’s milk (7% fat) and cream (64% fat) (Hårbølle Mejeri, Møn, Denmark) were mixed with the proportions being 30% and 70%, respectively. The dairy mixture was homogenized and heated to 95°C. Then, 10 mL ant water infusion (described above) was added for each kilogram of this dairy mixture, stirring until complete coagulation.^104^ The curd was cooled to 4°C for 6 h and strained through a cheesecloth. The mascarpone-like cheese was stored at 4°C.

##### Cocktail clarified with ant milk-wash

Milk-wash is a technique commonly used in cocktails to clarify a liquid. Milk Punch, the common drink prepared with this technique, is a dairy-based drink dating to the late 1600s and early 1700s. The earliest written recipe of clarified milk punch comes from a 1711 cookbook by Mary Rockett.^41^ To do so, the milk is curdled by lowering the pH, which makes it possible to strain out the dairy solids. The result is a clear beverage with more richness and body. It is also a popular modern technique at cocktail bars, normally produced in large amounts.^41^ Using this technique, a cocktail was developed, but by inducing the milk coagulation with dehydrated ants. The formic acid replaced other products commonly used to lower the pH, such as lemon, lime, or any other acids like citric or ascorbic acid. For the cocktail, an alcoholic base was made using 181 g of brandy (Ximenez Spinola Battonage Brandy, Spain), 101 g of genepi liqueur (Genepi Grand Tetras, France), and 118 g of apricot liqueur (Bitter Truth Apricot Liqueur, Germany) mixed in a glass jar. 10 g of dehydrated ants (*Formica rufa*) were added and infused for 2 h at RT. The alcoholic mix plus dehydrated ants were poured into a glass jar with 200 g of milk (5% fat), stirred, and kept at 2°C for 6 h to curdle the milk. Subsequently, the mixture was filtered through a coffee filter and kept at 2°C until serving. The cocktail was garnished with 4 frozen ants. The cocktail is in the “aperitif” style of cocktails.

#### Ant collection and processing for experimental yogurts

Experimental yogurts were made in spring, according to the tradition,^7^ and in autumn, to test the seasonality of the fermentation. We used the red wood ant *Formica polyctena* for microbiome characterization and experimental yogurt fermentation, and *F. rufa* for microbiome characterization. *F. polyctena* is the sister species of *F. rufa*, where the later was used in the ethnographic account and at the Alchemist R&D. *F. polyctena* worker ants were collected at the end of May and the start of October 2022*,* from a colony in Rude Skov, Denmark (55.8334720, 12.474938410). Similarly, worker ants from a colony of *F. rufa*, found in Bøllemosen, Denmark (55.827676, 12.534667), were collected at the end of May and late September 2023. Ants were taken without disturbing the mound structure, considering several species within the group have experienced species declines within Europe.^92^ Ants were gathered with sterilized equipment and kept on ice while transported to the lab.

*F. polyctena* worker ants were then processed to make the 3 elaborations of ant yogurt, live, frozen, and dehydrated. Live ants were provided sterile 15% sucrose as food and used to make ant yogurt within the week of collection. Prior to freezing, ants were aseptically removed from the nest material, then snap-frozen and stored at −20°C. Dehydrated ants were prepared by placing frozen ants in a vented Petri dish with sterile filter paper and incubated at 40°C for 16 h and were used within a week. Live and dehydrated ants were frozen at −20°C for later microbiome characterization.

The ant species were identified by barcoding of the *cytochrome c oxidase I gene (COXI)*. DNA was extracted with Chelex (Sigma Aldrich, Denmark), amplified, cleaned, and sequenced.^105^ PCRs were performed with primers LCO1490 5′-GGTCAACAAATCATAAAGATATTGG-3′and HCO2198 5′-TAAACTTCAGGRTGACCAAAAAATCA-3′.^93^ PCR products were cleaned with PureIT ExoZap (Amplicon, Denmark) and sequenced at Macrogen (the Netherlands). The genetic identities of the consensus sequences were determined to be *F. polyctena* and *F. rufa*. This also matched the respective polydomous and monodomous nest architecture observed at the field sites and characteristic of the different species.

#### Live, dehydrated, and frozen elaboration of experimental ant-yogurt

Ant yogurts were made under sterile conditions within a laminar flow hood, to assure that the ants were the sole source of microbes. The yogurt was made with 10% w/v reconstituted milk powder in distilled water (Sigma Aldrich, Denmark) and sterilized at 121°C for 5 min to avoid caramelization. Yogurts were made in Falcon tubes with 30 mL of sterile milk and 0.5 g live ants, crushed with a sterile pestle, 0.5 g frozen ants, or 0.4 g dehydrated ants. Yogurts and negative controls were incubated at 42°C for 8 h and then stored at 4°C overnight, according to standard yogurt-making practices.^9^^,^^68^ The following day yogurts were subsampled for microbial culturomics, acid quantification, DNA extraction, and proteomics. Subsamples were stored at −20°C.

In the spring, six samples of each of the live, frozen, and dehydrated ant-yogurts were made as well as 2 negative controls of milk alone, one incubated and one not incubated. In the autumn, the same design was used, but frozen ant-yogurt was not made as it was found to contain pathogens.

#### DNA extraction of yogurts and ants

DNA was extracted from the yogurts and ants to assess the microbial community composition. All extractions were performed under sterile conditions in a laminar air flow. The yogurt samples were first homogenized by vortexing, then passed through a Corning cell strainer with pore size 100 μm (Sigma Aldrich, Denmark) to remove ant body parts. The yogurt underwent three washes prior to lysis to remove excess fats that may act as inhibitors and to pellet microbial cells. First, 3 g of yogurt and 10 mL of sterile 2% sodium citrate (Sigma Aldrich, Denmark) solution pH 7.4 were incubated at room temperature for 10 min, then centrifuged at 5,000 g for 10 min at 4°C. Second, the pellet was retained and washed in 15 mL TES buffer (50 mM Tris, 1 mM EDTA, 20% sucrose) and centrifuged at 5,000 g for 10 min at 4°C. A final wash was done in 1 mL TES, which was pelleted, and supernatant removed. For the ants, two ants were used per DNA extraction. Prior to extraction, 200 μL molecular water was added to the ants and they were crushed with a sterile pestle.

DNA extraction was performed with the DNeasy Blood and Tissue Kit (Qiagen, Germany) according to manufacturer’s instructions for gram positive bacteria with the following modifications. Physical lysis of ants and yogurts was performed via bead-beating with 0.1 mm G2 DNA/RNA Enhancer beads (VWR, Denmark) in a TissueLyser (Qiagen, Denmark) at 30 Hz for 3 min. Samples then underwent chemical lysis, including 30 min lysis in lysozyme (Sigma Aldrich, Denmark) with an addition of 2 μL of mutanolysin (25 U/⎧L) (Sigma Aldrich, Denmark) followed by 1 h proteinase K lysis. The final elution of the DNA extract was 200 μL in AE buffer, passed twice through the column to maximize yield. Due to exceptionally low DNA concentrations in some experimental yogurts, three extractions were performed per sample, and thus 9 g of yogurt in total was washed, extracted, and pooled per sample. Similarly, two ant extractions were pooled for a final sample representing four ants. The elutes were pooled and concentrated with AMP Pure XP Reagent (Beckman Coulter, Denmark) according to manufacturer’s instructions, then underwent a clean-up for inhibitors with the DNeasy PowerClean Pro kit (Qiagen, Denmark) according to manufacturer’s instructions.

The final samples for sequencing included yogurts, ants, and several controls. Yogurt samples thus included 20 samples from spring and 13 from autumn, as previously described. From the ants, there were six final samples for live and for dehydrated *F. polyctena* each from spring and autumn, and six samples of live *F. rufa* in spring and autumn. In addition to the experimental yogurts and ants, a mock community of standard bacteria (Zymobiomics, Nordic Biosite, Denmark) was included to determine any community bias generated by extraction and later metabarcoding. Further, three conventional plain yogurts were included as positive controls. Last, five blank DNA extractions were included as negative controls for DNA extraction.

#### Quantification of bacterial load

The bacterial microbial loads of the yogurt, ant, and control samples were assessed with real-time PCR (qPCR). To quantify the bacteria in samples, the Femto Bacterial DNA Quantification Kit (Zymobiomics, Denmark) was used according to the manufacturer’s instructions. The Femto Bacterial Kit targets the 16S *rRNA* gene of bacteria and estimates the amount of bacterial DNA (ng/⎧L) in a sample based on a series of standards and non-template control. qPCR of samples was run in duplicate on Mx3005p (Agilent, Germany). The technical replicates demonstrated minimal variation and were thus averaged. Subsequently, the amount of DNA/mL of yogurt, in the total yogurt, or ant starter, was back calculated based on the standards and experimental parameters.

#### Bacterial community metabarcoding

The bacterial community of the ants, yogurts, and controls was characterized with bacterial metabarcoding. Library preparation and targeted sequencing of the 16S *rRNA* V3-V4 region was performed with the primers 341F (5’ -CCTAYGGGRBGCASCAG- 3′) and 806R (5’ -GGACTACNNGGGTATCTAAT- 3′) at Novogene Europe (Cambridge, United Kingdom). In brief, amplicons for the targeted region underwent size selection, then equivalent amounts of PCR product were end-repaired, A-tailed, and ligated with Illumina adapters. Library concentrations were checked with Qubit and qPCR, and size distribution was checked with BioAnalyzer. Libraries were pooled and sequenced on Illumina for pair-ended 250 bp at a depth of 30k per sample. After sequencing, primers and adaptors were removed by Novogene, and raw reads were provided for analysis.

#### Bacterial culturomics of milk ferments and ants

Bacteria from the ant yogurts and ants were cultured and identified with Sanger sequencing. 100 μL of yogurt sample was inoculated and plated in De Man, Rogosa and Sharpe (MRS) (VWR Chemicals, US), Gifu Anaerobic Medium, modified (GAM) (HyServe GmbH & Co. KG, Germany), yeast peptone dextrose (YPD), and Luria-Bertani (LB) media. YPD contained 10 g/L yeast extract, 20 g/L peptone, and 20 g/L D-glucose. LB contained 10 g/L peptone, 5 g/L yeast extract and 0.5 g/L sodium chloride. A total of 20 g/L agar was added for solid cultivations. For ants, individual ants were inoculated into liquid media. Tubes and plates were incubated at 30°Cover 2 days, or until growth was visible. All four media were cultivated in aerobic conditions and MRS and GAM were also incubated anaerobically. Subcultures were plated and incubated in the same conditions. Distinct bacterial colonies from plates were identified with Sanger sequencing of the conserved 16S *rRNA* gene, which included colony PCR (cPCR) with primers 5′-AGAGTTTGATCCTGGCTCAG-3′ and 5′-CCGTCAATTCCTTTRAGTTT-3′.^94^ All cPCR reactions consisted of 12.5 μL of Red-Taq Mastermix (VWR, Denmark), 0.5 μL of forward 10 μM primer, 0.5 μL of reverse 10 μM primer, 11.5 μL of water and a bacterial colony. The reactions followed the same thermocycler program that consisted of an initial 4 min at 94°C, followed by 35 cycles of 94°C for 30 s, 51°C for 30 s, 72°C for 1 min with a final 72°C for 10 min. PCR products were Sanger sequenced (Eurofins Scientific SE).

#### Metabolic characterization of *Fructilactobacillus* sanfranciscensis

*F. sanfranciscensis* was characterised with two standard carbon substrate arrays, PM1 and PM2 according to manufacturer’s instructions for species of *Lactobacillus*. In brief, the inoculum for the Biolog substrate arrays (65%T, IF-0a GN with Dye mix G and PM1 or PM2 supplement; 12xstock) was prepared using freshly growth overnight cultures of *F. sanfranciscensis* on MRS. Data was recorded for 36 h at 33°C using the Omnilog system (Biolog, Hayward, California, USA). The system recorded catabolism of substrates which was analyzed using the Omnilog PM software packages. Here, all data was normalized to the negative control (well A1). Only substrates where catabolism by *F. sanfranciscensis* was found were visualized in GraphPad Prisim.

#### pH, lactic, acetic, and formic acid measurement of yogurts

The pH and coagulation of all yogurts were assessed before and after fermentation. After fermentation, the pH was measured from one sample of each of the groups, and the coagulation of the treatment group was assessed. Given the abundance of formic acid found in *Formica* ants,^66^ we also examined the effect of formic acid on coagulation. Formic acid (Sigma Aldrich, Denmark) was added to the sterile milk until pH 4.6, 5.15, 5.9, and 6.6, which represented the range of pHs observed in conventional yogurts, ant yogurts, and sterile milk. The pH of the negative control was also measured. Additionally, pH of all the samples was measured prior to DNA extraction and enzyme analysis, after they had undergone a cycle of freezing and thawing. This was performed on a separate pH reader than that which provided the initial measurement before and after fermentation.

The amount of formic, lactic, and acetic acid in the samples was quantified with high-performance liquid chromatography (HPLC). Yogurt samples were centrifuged at 5000 rpm for 5 min at 4°C, and their supernatant was passed through a 0.2 ⎧m syringe filter (Sigma Aldrich, Denmark). The filtrate was analyzed by a Dionex Ultimate 3000 HPLC system (Thermo Fisher Scientific, USA) equipped with a refractive index detector and a Bio-Rad Aminex HPx87 column (Bio-Rad, USA). The column oven temperature was set at 30°C. The flow rate was set at 0.6 mL/min with 5 mM H_2_SO_4_ (Sigma Aldrich, Denmark) as an eluent. The injection volume was 10 ⎧L.

#### Proteomics of ant yogurts

Sample preparation for proteomics analysis was performed directly from the supernatant of the yogurt samples after centrifugation for 10 min at 14,000 g and further processed as described earlier.^106^ After centrifugation, the supernatants were subjected to the bicinchoninic acid (BCA) assay to estimate protein concentrations. Trypsin and LysC digestion mix (Promega) was added to 20 ⎧g protein of each sample and incubated for 8 h. Trifluoroacetic acid was added to halt digestion and the samples were desalted using C18 resin (Empore, 3M) before HPLC-MS analysis.

HPLC-MS analysis of the samples was performed on an Orbitrap Exploris 480 instrument (Thermo Fisher Scientific) preceded by an EASY-nLC 1200 HPLC system (Thermo Fisher Scientific). For each sample, 1 μg of peptides was captured on a 2 cm C18 trap column (Thermo Fisher 164946). Subsequently separation was executed using a 70 min gradient from 8% (v/v) to 48% (v/v) of acetonitrile in 0.1% (v/v) formic acid on a 15-cm C18 reverse-phase analytical column (Thermo EasySpray ES904) at a flow rate of 250 nL/min. For data-independent acquisition, the mass spectrometer was run with the HRMS1 method^107^ preceded by the FAIMS Pro Interface (Thermo Fisher Scientific) with a compensation voltage (CV) of −45 V. Full MS1 spectra were collected at a resolution of 120,000 and scan range of 400-1,000 m/z, with the maximum injection time set to auto. MS2 spectra were obtained at a resolution of 60,000, with the maximum injection time set to auto and the collision energy set to 32. Each cycle consisted of three DIA experiments each covering a range of 200 m/z with a window size of 6 m/z and a 1 m/z overlap, while a full MS scan was obtained in between experiments.

### Quantification and statistical analysis

#### Bacterial community analysis

The analysis of sequences was carried out in R (v. 4.3.1),^95^ which included determination of amplicon sequence variants (ASVs), taxonomic assignment, visualization, and statistical analyses. The dada2 pipeline (v. 1.30.0)^96^ was used for ASV and taxonomic assignment. Reads were not trimmed due to the overall high quality of sequences, but were filtered with the default parameters, with *maxEE* adjusted to (2,4). Reads were then paired with *mergePairs*, *minOverlap* set to 16, and chimeras were removed. Taxonomy was assigned to the species level where possible with the SILVA database (v. 138.1).^97^ Potential contaminants were then removed using SCRuB (v.0.0.1)^98^ based on the blank negative controls that were successfully sequenced. Mitochondria, chloroplasts, and Eukarya were removed, resulting in 63.87% of ASVs with genus-level classification and 82.23% with family-level. The mock community was then assessed to determine any potential bias created by DNA extraction and sequencing. All eight bacterial genera present in the mock community were validated.

The metabarcoding data was then analyzed and visualized using the packages phyloseq (v.1.46.0),^99^ microviz (v.0.12.0),^100^ and ggplot2 (v.3.4.4).^101^ The composition of the microbiome of ants and yogurts was visualized with microviz (Figures 3A and 3C). The beta diversity of the yogurts was visualized with phyloseq through an NMDS ordination of their Bray-Curtis distances, resulting in a plot ordinated by yogurt treatments (Figure 3D). The alpha diversity of the yogurts was further determined with phyloseq (Figure 3E). Last, the microbial loads of Lactobacillaceae and Bacillaceae in the ant starters and yogurts were estimated by multiplying the relative abundance of each genus, based on the metabarcoding data, by the quantified bacterial load, based on 16S qPCR. This was visualized using ggplot2 (Figures 4A and 4D).

We assessed the effect of treatment (live, dehydrated, or frozen) on the beta diversity of the yogurts using Bray-Curtis distances and an adonis PERMANOVA using the package vegan (v.2.6.4)^102^ and function *adonis2* (results). Subsequently, we performed a pairwise-comparison between the three treatments based on the PERMANOVA using the package pairwiseAdonis (v.0.4.1),^103^ which performs *p*-value adjustment for multiple comparisons (results). The alpha diversity of the three yogurts was based on the Shannon index. We performed a general linear model to determine the effect of treatment on the alpha diversity, confirming the parametric assumptions of the model (results). Pairwise comparisons were then performed with a Tukey HSD (Figure 3 legend). Pairwise comparisons of the Lactobacillaceae and Bacillaceae load in the ant starters and yogurts was performed with two sample t-tests for equal or unequal variance depending upon the data (results; Figure 4 legend).

#### Sanger sequencing analysis of ants and isolated bacteria

Sequences were trimmed and aligned using default settings in Geneious (Biomatters Ltd., New Zealand), and the consensus sequence was compared to NCBI’s nucleotide database with BLASTn. For bacterial isolates, results were presented in a Sankey diagram (app.rawgraphs.io) (Figures 4B and 4E).

#### Acid quantification and analysis from HPLC for yogurts

Peaks were identified by comparison to the prepared standards, and integration of the peak areas was used to quantify acids from obtained standard curves using the software Chromeleon7 (Thermo Fisher Scientific, USA). Plots were visualised in GraphPad Prism (Figure 5A). T-tests were used to compare organic acid levels between samples, with Benjamini-Hochberg correction for multiple testing (results).

#### Analysis of proteases and peptidases in ant yogurts

To assign the proteases to their potential organisms of origin, we created a database consisting of the proteases, peptidases and proteins based on UniProt reference proteomes, the gold standard in proteomics. The proteomes consisted of the ant host, bacteria from the ant yogurt, and two conventional yogurt bacteria. For the ant, we used the *F. exsecta* proteome, since the *F. polyctena* proteome was not publicly available, and *F. exsecta* is a closely related species. From the *F. exsecta* proteome, we selected the proteases specifically. For all the bacteria included, we refined our database to only include proteases and peptidases in the proteome. We selected bacteria most prevalent among the bacteria isolated from the live ant yogurt bacterial metabarcoding and culturomics: *Fructilactobacillus sanfranciscensis*, *Oecophyllibacter saccharovorans*, and *Paenibacillus* sp. We also included *Wolbachia pipentis*, which is closely related to the obligate intracellular bacteria found in *Formica* ants^52^ and is represented in our analysis. We included the conventional yogurt bacteria *Streptococcus thermophilus* and *Lactobacillus delbrueckii* subsp. *bulgaricus. Streptococcus* was only present in low abundance in the bacteria metabarcoding data, and *L. delbrueckii* subsp. *bulgaricus* was isolated in one instance. We included them to determine if the ant yogurt contained proteases with the same or similar functionality. Last, we included common milk proteins, such as caseins, found in the *Bos taurus* proteome. For DIA data analysis, Spectronaut v18 (Biognosys, Switzerland) was used for protein identification and relative quantification of peptides. The default settings for “directDIA” were applied with an FDR cut-off of 1%, except for MS1 quantification for the peptides. Protein abundances were inferred from the peptide abundances using the MaxLFQ algorithm available within Spectronaut.

In the analysis of the results, first we determined if proteases, peptides, and milk proteins, changed in relative abundance between spring or autumn and live or dehydrated samples. We were unable to determine the abundance of a subset of proteases identified, likely due to their low abundance, and they were therefore not included in the analyses. For each protein, the average relative abundance was computed across all autumn samples and all spring samples. Similarly, the average relative abundance was computed across all live samples and all dehydrated samples. The log2 fold change (log2FC) was calculated.

*p*-values were obtained by comparing the relative abundances of each protein across all samples and their replicates in the autumn versus spring conditions. Similarly, *p*-values were calculated by comparing the relative abundances of each protein across all samples and their replicates in the alive versus dehydrated conditions. The Benjamini-Hochberg procedure was applied to adjust the *p*-values for multiple comparisons, controlling the false discovery rate (FDR). The adjusted *p*-values were then transformed to their -log10 values for further analysis and visualization (Table S2).

Second, we visualized the composition of the proteases and peptidases in GraphPad Prism (Figure 5B). To do so, we removed the milk proteins originating from *Bos taurus*, given they made up a high proportion of the proteome. The aim was thus to clarify ant or bacterial contributions to the enzymatic profile. The relative abundance of each protease or peptidase visualized was thus based on the total abundance of proteases and peptidases in each sample, and not the proteome.

## Supplemental information

Document S1. Figures S1–S7

Table S1. pH of yogurts and formic acid controls before and after incubation, related to Figure 5

Table S2. Absolute abundance of milk proteins, proteases, and peptidases in proteomes, related to Figure 5
